# Self-reported smoking cessation activities among Swiss primary care physicians

**DOI:** 10.1186/1471-2296-10-22

**Published:** 2009-03-25

**Authors:** Isabelle Jacot Sadowski, Christiane Ruffieux, Jacques Cornuz

**Affiliations:** 1 Department of Ambulatory Care and Community Medicine, University of Lausanne, Switzerland; 2 Institute of Social and Preventive Medicine (IUMSP), University of Lausanne, Switzerland

## Abstract

**Background:**

Individual counselling, pharmacotherapy, and group therapy are evidence-based interventions that help patients stop smoking. Acupuncture, hypnosis, and relaxation have no demonstrated efficacy on smoking cessation, whereas self-help material may only have a small benefit. The purpose of this study is to assess physicians' current clinical practice regarding smokers motivated to stop smoking.

**Methods:**

The survey included 3385 Swiss primary care physicians. Self-reported use of nine smoking cessation interventions was scored. One point was given for each positive answer about practicing interventions with demonstrated efficacy, i.e. nicotine replacement therapy, bupropion, counselling, group therapy, and smoking cessation specialist. No points were given for the recommendation of acupuncture, hypnosis, relaxation, and self-help material. Multivariable logistic analysis was performed to identify factors associated with a good practice score, defined as ≥ 2.

**Results:**

The response rate was 55%. Respondents were predominately over the age of 40 years (88%), male (79%), and resided in urban areas (74%). Seventeen percent reported being smokers. Most of the physicians prescribed nicotine replacement therapy (84%), bupropion (65%), or provided counselling (70%). A minority of physicians recommended acupuncture (26%), hypnosis (8%), relaxation (7%), or self-help material (24%). A good practice score was obtained by 85% of respondents. Having attended a smoking cessation-training program was the only significant predictor of a good practice score (odds ratio: 6.24, 95% CI 1.95–20.04).

**Conclusion:**

The majority of respondents practice recommended smoking cessation interventions. However, there is room for improvement and implementing an evidence-based smoking cessation-training program could provide additional benefit.

## Background

Physicians play an important role in smoking cessation, and medical advice and pharmacotherapy are effective clinical interventions to prompt and help patients to stop smoking [1-4]. Brief physician advice is effective for successful smoking cessation with odds ratio (OR) 1.66 (95% CI 1.42 to 1.94) versus no advice or usual care [1]. The risk ratio (RR) of abstinence for any form of nicotine replacement therapy (NRT) relative to placebo or non-NRT group is 1.58 (95% CI 1.50 to 1.66) [3]. Bupropion double the odds of cessation (OR 1.94, 95% CI 1.72 to 2.19) [4]. Group therapy is also effective (OR 2.19, 95% CI 1.42 to 3.37 versus no intervention controls) whereas acupuncture, hypnosis, and autogenic training (relaxation technique with self-hypnosis) have no demonstrated efficacy on smoking cessation [2,5-7]. Self-help material such as brochures on tobacco and health might increase quit rates, but the size of the effect is small. Self-help materials have no additional benefit when used in concert with advice from a healthcare professional or with nicotine replacement therapy [8].

Around 80% of the Swiss general population visits a doctor annually, and most patients expect smoking cessation interventions from their physician [9-11]. Furthermore, studies have found clinical activities related to smoking cessation to be highly cost-effective in developed countries, particularly when compared to other currently accepted preventive activities, such as treating mild to moderate hypertension and lowering cholesterol levels [12].

However, some doctors perceive barriers to engaging in smoking cessation counselling, such as lack of patient interest, lack of confidence in their ability to discuss smoking cessation, lack of time, fear of harming the doctor-patient relationship and difficulty to advice smokers without smoking-related problems [13-16]. When practitioners are asked to judge the importance of different preventive activities in their clinical practice, 93% judge controlling blood pressure to be "very important"; in contrast, only 73% find counselling on smoking cessation to be "very important" [13].

Physicians' own health habits, like sedentary lifestyle or drinking more than three alcoholic drinks per day, could also negatively influence their attitudes toward smoking cessation counselling (smoking status was not a predictor in this study) [13].

Surveys of Swiss patients have reported that 81% to 88% of smokers have discussed their smoking habits with a physician, but only 34% were advised to stop [17,18].

In 2002, when the survey was conducted, the national program for smoking prevention 2001–2005 has just started (Federal Office of Public Health). An educational program to improve physicians' skills and effectiveness in smoking cessation has been developed and assessed by residents in outpatients clinics few years before [19]. Twenty-five teachers were trained in 2002 in order to disseminate the smoking cessation program nationwide. A reference manual on smoking cessation in clinical practice including algorithms for smokers approach and therapy, as well as patients' brochures were available. There were however hardly any smoking cessation specialists, except in few University hospitals.

When the survey took place, smoking cessation post-graduate training sessions were not uniformly implemented throughout Switzerland with linguistic regions difference: there were proportionally more training sessions in French-speaking region than in German part.

The purpose of this survey was to assess clinical practice of Swiss primary care physicians toward patients motivated to stop smoking. Our ultimate objective was to highlight areas in which tobacco prevention programs should focus their attention in order to enhance delivery of evidence-based clinical practice for promoting smoking cessation.

## Methods

### Data collection

Using the national registry of the Swiss primary care physicians, we randomly sampled 50% (n = 1872) of the physicians in the German-speaking cantons, and included all of the physicians in the French-speaking (n = 1286) and Italian-speaking (n = 227) regions. An invitation announcing the purpose of the survey and a copy of the questionnaire (with a self-addressed, pre-stamped envelope) were mailed to all 3385 doctors. Non-respondents received a single reminder letter eight weeks later that included a copy of the questionnaire. Data were processed anonymously.

The questionnaire was developed in French and 18 colleagues in Lausanne, Switzerland, conducted pilot testing. The instrument was professionally translated into German and Italian and checked for accuracy prior to study launch.

### Statistical analyses

Univariate analyses were performed to describe the data. The multivariate model used one set of dichotomous data that combined information about the self-reported use of nine tobacco cessation interventions for smoking patients who wanted to quit, as described below.

For scoring the smoking cessation practice of physicians, one point was given for each positive answer about using the following evidence-based interventions: (a) prescription of nicotine replacement therapy, (b) prescription of bupropion, (c) direct smoking cessation counselling, (d) recommendation to attend group smoking cessation therapy, and (e) referral to a smoking cessation specialist for counselling. No points were added or deducted for the reported use of (a) acupuncture, (b) hypnosis, (c) relaxation techniques, or (d) written self-help material. Possible scores ranged from 0 to +5. We predefined a dichotomous variable indicating a score ≥ 2, to be interpreted as an indicator of good physician practice.

A multivariable logistic analysis was performed to identify factors favouring good physician practice. The following variables were considered: previously attending smoking cessation training, years since graduation (defined as ≤ 20 years versus > 20 years), urban residence, and smoking status (defined as daily smoker versus non-smoker including ex-smoker and occasional smoker). For this analysis, data were weighted according to the sample size. All statistical analyses were performed using Stata (StataCorp, College Station, TX, USA).

## Results

### Sample characteristics

1856 physicians, for a response rate of 54.8%, returned the questionnaires. The response rates were 54.5%, 54.8%, and 57.7% for German-, French-, and Italian-speaking doctors, respectively (p = 0.654).

Respondents were predominately 40 years of age or older (88%), male (79%), and practiced in urban areas (74%). Over half of the sample came from the German-speaking area, a third from French-speaking regions, and less than a tenth of respondents were Swiss-Italian. The majority of respondents graduated between 11 and 30 years before to the survey, 5% within the last 10 years of the survey, and 12% over 30 years before the survey. Only 5% of physicians had received training on smoking cessation counselling with significant linguistic difference (German:1.5%, French: 10.5%, Italian: 3.2%, p < 0.001). Slightly over one-sixth reported being current daily or occasional smokers (Table 1).

**Table 1:** Participants characteristics (n= 1856)

Variable		**%**
Age	< 40 years	12.0
	40–49 years	39.3
	> 49 years	48.7

Male gender		78.8

Linguistic region	German	55%
	French	38%
	Italian	7%

Urban residence		73.6

Years since graduation from medical school	0–10 years	4.7
	11–20 years	32.0
	21–30 years	51.6
	30–40 years	11.7

Prior attending smoking cessation training		5.1

Smoking status	Daily smoker	7.2
	Occasional smoker	10.4

Regarding physicians' choice of evidence-based smoking cessation interventions, the majority reported prescribing nicotine replacement therapy (84%), bupropion (65%), or providing an individual consultation to patients on their tobacco use (70%). About one-third reported recommending participation in a smoking cessation group, and one-tenth referred smokers to a smoking cessation clinic (Table 2).

**Table 2:** Physicians' self-reported use of smoking cessation interventions techniques for patients motivated to stop smoking (n = 1853)

Physicians self-reported practices		%
Demonstrated efficacy	Prescription of nicotine replacement therapy	84.0
	Prescription of bupropion	65.4
	Personal consultation on patient's tobacco use	70.1
	Recommendation to attend smoking cessation groups	29.2
	Referral to a smoking cessation specialist	9.6

Efficacy not yet shown	Acupuncture	26.1
	Hypnosis	7.9
	Relaxation	7.4
	Brochures	23.7

Regarding the unproven techniques, one-fourth of physicians recommended acupuncture and under one-tenth proposed hypnosis or relaxation techniques. Nearly, one-fourth provided brochures on the topic.

**Figure 1:**
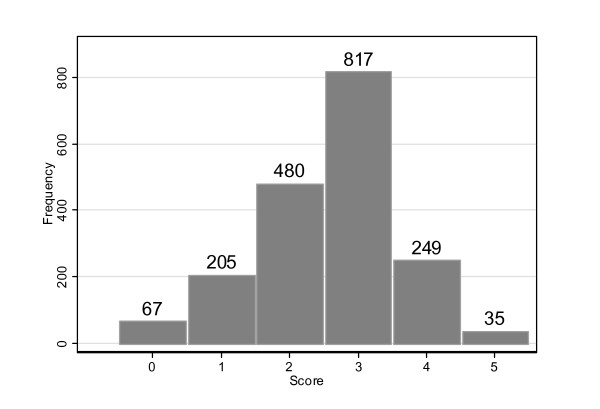
**Distribution of smoking cessation clinical practice scores of Swiss physicians (n = 1853)**.

Using the formulas described in the Methods section, 85% of physicians (n = 1581) had a "good" score on tobacco cessation practices (Figure 1).

### Predictors of clinical tobacco cessation practices

Our model for predicting characteristics of physicians with a "good" clinical practice score included having received smoking cessation training, having received their medical diploma more than 20 years prior to the survey, practicing in an urban environment, and personal smoking status. The language region (French, German, or Italian) was not included in the model because of its interaction with the availability of smoking cessation courses for physicians, with less training sessions in the German part of Switzerland. Gender was not included in our model because this information was not needed to plan future programs.

Having received smoking cessation training was the only statistically significant predictor of a "good" clinical practice score (Table 3).

**Table 3:** Predictors of smoking cessation clinical practices: Multivariable logistic analysis.

Outcome	Predictor	Odds ratio (95% CI)	P
Good score	Prior attending smoking cessation training	6.24 (1.95, 20.04)	0.002
	Graduation >20 years	1.13 (0.84, 1.51)	0.415
	Urban residence	0.75 (0.54, 1.06)	0.103
	Daily smoker	0.87 (0.51, 1.48)	0.597

Adjusted odds ratio.

## Discussion

The results of this survey suggest that the majority of Swiss primary care physicians practice evidence-based smoking cessation interventions. A large majority of these physicians reported prescribing first-line therapies including nicotine substitutes and bupropion, as well as providing individual counselling on how to quit using tobacco. Only a small minority reported recommending attending a smoking cessation group or referring smokers to tobacco specialists.

The only characteristic positively associated with a good practice score on smoking cessation intervention was having attended smoking cessation training. Physicians' own smoking status was not significantly correlated with smoking cessation practices, as has been reported in other studies; however, an association between personal health behaviour and attitude toward health promotion has been reported [13,20,21]. The interval since physicians received their medical diplomas and whether or not they practiced in an urban environment were not significantly correlated with tobacco cessation interventions.

International studies have assessed physicians' incorporation of tobacco control interventions in their clinical practice and their opinions about national guidelines [22-25]. However, it was not possible to compare Swiss physician practices with this data because the questions in the surveys and the health systems are not similar.

Our study had some limitations. Firstly, our response rate (55%) led us to question whether there was potential bias. However, this participation rate is in concordance with rates reported in other physician surveys [21-23]. Furthermore, response bias might not pose as much of a methodological challenge in this relatively homogenous population in terms of age and gender as compared to the general physician population, except the slight smaller proportion of respondents who were daily smokers, 7% versus 12% reported in a previous survey [26]. Secondly, respondents were perhaps more motivated in this topic, that could have resulted in overestimation of the quality of the smoking cessations interventions. A third limitation was using the score variables to combine responses to multiple survey questions. These unvalidated measures could have led to bias in the regression analyses. Giving equal weighting to the interventions is an approximate way to establish the score, as recommending counselling is probably a weaker intervention than direct counselling.

Just self-reported attitude, no measure of frequency, no measure of whether physicians assessed smoking status and non-uniformly implementation of smoking cessation training sessions with linguistic difference are additional limitations.

We think there are important policy implications in improving the practice scores among physicians who had or had not previously taken a smoking cessation course. Although this survey did not measure clinical outcomes in patients, we assumed that patients being followed by physicians with better smoking cessation practice scores would have higher quit rates. Therefore, our data provide impetus for promoting more smoking cessation training courses for physicians. Further evidence of the benefits of training physicians can be found in a prior randomized controlled trial among physicians, in which patients assigned to doctors who received smoking cessation training had improved smoking-related behavioural and clinical outcomes [27].

Since this survey was conducted in 2002, the Swiss national program for tobacco prevention published clinical recommendations and organized smoking cessation training courses for physicians [28-31]. More than 4000 Swiss physicians have provided teaching assistance.

## Conclusion

The majority of participating Swiss physicians practice recommended smoking cessation interventions with patients motivated to stop smoking. However, the number of physicians practicing these interventions could be increased and implementation of the interventions could be improved; smoking cessation training courses are an effective means of achieving both goals.

## Competing interests

JC is member of the Swiss expert panel that promotes smoking cessation and is in charge of the Swiss physician smoking cessation-training program "Vivre sans tabac". IJS is collaborating in the Swiss physician smoking cessation-training program "Vivre sans tabac".

## Authors' contributions

IJS participated to the interpretation of data and drafted the manuscript. CR participated to the study design and performed the statistical analysis. JC participated to the study design, interpretation of data and revised the manuscript. All authors read and approved the final manuscript.

## Sources of funding for the study

Department of Ambulatory Care and Community Medicine, Lausanne University Hospital, Switzerland. Institute of Social and Preventive Medicine (IUMSP), University of Lausanne, Switzerland.

## Pre-publication history

The pre-publication history for this paper can be accessed here:


